# Life expectancy, healthy life expectancy, and burden of disease in older people in the Americas, 1990–2019: a population-based study

**DOI:** 10.26633/RPSP.2021.114

**Published:** 2021-09-30

**Authors:** Ramon Martinez, Patricia Morsch, Patricia Soliz, Carolina Hommes, Pedro Ordunez, Enrique Vega

**Affiliations:** 1 Pan American Health Organization Washington D.C. United States of America Pan American Health Organization, Washington D.C., United States of America

**Keywords:** Health of the elderly, mortality, morbidity, Americas, Salud del anciano, mortalidad, morbilidad, Américas, Saúde do idoso, mortalidade, morbidade, América

## Abstract

**Objective.:**

To describe the life expectancy, healthy life expectancy, disease burden, and leading causes of mortality and disability in adults aged 65 years and older in the Region of the Americas from 1990 to 2019.

**Methods.:**

We used estimates from the Global Burden of Disease Study 2019 to examine the level and trends of life expectancy, healthy life expectancy, years of life lost, years lived with disability, and disability-adjusted life years (DALYs).

**Results.:**

Across the Region, life expectancy at 65 years increased from 17.1 years (95% uncertainty intervals (UI): 17.0–17.1) in 1990 to 19.2 years (95% UI: 18.9–19.4) in 2019 while healthy life expectancy increased from 12.2 years (95% UI: 10.9–12.4) to 13.6 years (95% UI: 12.2–14.9). All-cause DALY rates decreased in each older persons’ age group; however, absolute proportional DALYs increased from 22% to 32%. Ischemic heart disease, stroke, and chronic obstructive pulmonary disease were the leading causes of premature mortality. Diabetes mellitus, age-related and other hearing loss, and lower back pain were the leading causes of disability.

**Conclusion.:**

The increase in life expectancy and decrease of DALYs indicate the positive effect of improvements in social conditions and health policies. However, the smaller increase in healthy life expectancy suggests that, despite living longer, people spend a substantial amount of time in their old age with disability and illness. Preventable and controllable diseases account for most of the disease burden in older adults in the Americas. Society-wide and life-course approaches, and adequate health services are needed to respond to the health needs of older people in the Region.

## RESUMEN

**Objetivo.:**

Describir la esperanza de vida, la esperanza de vida saludable, la carga de enfermedad y las principales causas de mortalidad y discapacidad en personas adultas de 65 años o más en la Región de las Américas desde 1990 hasta el 2019.

**Métodos.:**

Se emplearon estimaciones del estudio sobre la carga mundial de enfermedad del 2019 para examinar las tendencias y el nivel de la esperanza de vida, la esperanza de vida saludable, los años de vida perdidos, los años vividos con discapacidad y los años de vida ajustados en función de la discapacidad (AVAD).

**Resultados.:**

En toda la Región, la esperanza de vida a los 65 años aumentó de 17,1 años (intervalos de incertidumbre [IU] del 95 %: 17,0–17,1) en 1990 a 19,2 años (IU del 95 %: 18,9–19,4) en el 2019, mientras que la esperanza de vida saludable se incrementó de 12,2 años (IU del 95 %: 10,9-12,4) a 13,6 años (IU del 95 %: 12,2–14,9). Las tasas de AVAD debida a cualquier causa disminuyeron en cada grupo etario de mayor edad; sin embargo, los AVAD absolutos proporcionales aumentaron de 22 % a 32 %. La cardiopatía isquémica, los accidentes cerebrovasculares y la enfermedad pulmonar obstructiva crónica fueron las principales causas de muerte prematura. La diabetes mellitus, la pérdida de la audición relacionada con la edad y de otro tipo, y la lumbalgia fueron las principales causas de discapacidad.

**Conclusiones.:**

El aumento de la esperanza de vida y la disminución de los AVAD indican el efecto positivo de las mejoras de las condiciones sociales y las políticas de salud. Sin embargo, el menor aumento de la esperanza de vida saludable indica que, a pesar de vivir más tiempo, las personas pasan una parte sustancial de su vejez con discapacidades y enfermedades. Las enfermedades controlables y prevenibles representan la mayor parte de la carga de enfermedad de las personas mayores en la Región. Se requieren enfoques a escala de toda la sociedad y el curso de vida, y servicios de salud adecuados para responder a las necesidades de salud de las personas mayores en la Región.

## RESUMO

**Objetivo.:**

Descrever a expectativa de vida, a expectativa de vida saudável, a carga de doenças e as principais causas de mortalidade e incapacidade em adultos a partir dos 65 anos de idade na Região das Américas de 1990 a 2019.

**Métodos.:**

Utilizamos estimativas do Estudo de Carga Global da Doença 2019 para examinar o nível e as tendências da expectativa de vida, expectativa de vida saudável, anos de vida perdidos, anos vividos com incapacidade e anos de vida ajustados por incapacidade (AVAI).

**Resultados.:**

Em toda a Região, a expectativa de vida aos 65 anos aumentou de 17,1 anos (intervalos de incerteza (II) de 95%: 17,0-17,1) em 1990 para 19,2 anos (II de 95%: 18,9-19,4) em 2019, enquanto a expectativa de vida saudável aumentou de 12,2 anos (II de 95%: 10,9-12,4) para 13,6 anos (II de 95%: 12,2-14,9). As taxas de AVAI por todas as causas diminuiu em todos os grupos de pessoas idosas; porém, em termos absolutos, os AVAI proporcionais aumentaram de 22% para 32%. A cardiopatia isquêmica, o acidente vascular cerebral e a doença pulmonar obstrutiva crônica foram as principais causas de mortalidade precoce. A diabetes melitus, a perda da audição – em função da idade ou por outros motivos – e a dor lombar foram as principais causas de incapacidade.

**Conclusão.:**

O aumento da expectativa de vida e a diminuição dos AVAI indicam o impacto positivo das melhorias nas condições sociais e nas políticas de saúde. Porém, o menor aumento na expectativa de vida saudável indica que, apesar de viverem mais, as pessoas passam uma quantidade considerável de tempo na velhice com incapacidade e doença. As doenças preveníveis e controláveis representam a maior parte da carga de doença nas pessoas idosas nas Américas. Abordagens que afetem a sociedade como um todo e o curso de vida, e serviços de saúde adequados, são necessários para atender às necessidades de saúde das pessoas idosas na Região.

Living longer has been one of humanity’s greatest ambitions, and currently living more than 80 years is a realistic expectation in many countries (1). Life expectancy captures mortality along the entire life course (2) and its improvement is positively associated with welfare and health. In 2019, life expectancy at birth reached 73.3 years globally but with a difference of around 16 years between high-income and low-income countries (3). Moreover, living longer and healthier is essential to achieving the 2030 Agenda for Sustainable Development (4). Certainly, healthy life expectancy indicates how much a country has reduced the incidence, duration, and severity of major diseases. Healthy life expectancy is also strongly associated with socioeconomic level and access to and quality of health care. Life expectancy and healthy life expectancy at 65 years reflect the level of well-being, health, and health care that a given society can offer to those who survive into older adulthood.

In 2018, for the first time, people aged 65 years and older outnumbered children under-5 years globally (1). The reductions in highly prevalent infectious diseases, infant mortality and fertility, together with increased human longevity have led to fast population aging. Moreover, rapid urbanization and the increase in unhealthy lifestyles, among other determinants, have accelerated the epidemiological transition and fueled the epidemic of noncommunicable diseases (NCDs) (5, 6).

Globally, NCDs accounted for 41.1 million deaths in 2017, representing 73.5% of total deaths and over 80.0% of deaths at age 60 years and older. In addition, avoidable NCDs accounted for 83.9% of all NCD deaths worldwide (7). The effect of NCDs is higher and more extensive in low- and middle-income countries. In 2011, in response to the global NCD epidemic and population aging, the United Nations General Assembly called for countries to tackle NCDs and their risk factors (8). In 2020, the General Assembly declared 2021–2030 the Decade of Healthy Ageing (9, 10).

Understanding the magnitude of and trends in the disease burden in older people and the underlying conditions driving those trends is key for designing effective strategies to improve the health of older people, prioritizing interventions, allocating resources and monitoring progress (11). A comprehensive study of the burden of diseases among older people is needed for the Region of the Americas, where the rise in aging populations is accelerating (12). For example, people older than 60 years old make up about 21% of the total population in Canada and the United States of America (USA), 13% in the Caribbean, 12% in South America, and 9% in Central America (13). Wide social and health disparities accompany this transition, and NCDs are shaping the pattern of morbidity and mortality (14).

This study aimed to describe life expectancy, healthy life expectancy, and burden of diseases in adults aged 65 years and older in the Region of the Americas from 1990 to 2019, and identify the leading causes of mortality and disability to inform programmatic and policy development.

## METHODS

We examined the level, distribution, and trends of life expectancy and healthy life expectancy, and disease burden in older adults aged 65 years and older (65+ years) in the Americas from 1990 to 2019. We used estimates from the 2019 Global Burden of Diseases, Injuries and Risk Factors Study (GBD study) (15). The GBD study is a comprehensive, multinational epidemiological study that estimates disease burden for every country in the world. It is an ongoing effort, updated annually, and is designed to allow consistent comparison over time from 1990 to 2019, by age and sex, and across locations. The study produces standard epidemiological measures such as incidence, prevalence, deaths, and summary measures of health loss for 369 diseases and injuries (16). The 2019 GBD study complies with the Guidelines for Accurate and Transparent Health Estimates Reporting (GATHER) (17). The methods and data sources used in the GBD 2019 have been published elsewhere (3, 15, 18) and are summarized in the appendix.

### Health expectancy and health loss outcome measures and data source

We examined the level, distribution, and trends of health expectancy measures, including life expectancy, healthy life expectancy, and years of life spent with poor health among people aged 65+ years by age, sex, and location from 1990 to 2019. Life expectancy is the number of years a person is expected to live at any given age. The methods to obtain life expectancy are reported elsewhere (19). Healthy life expectancy is the average number of years of life spent in good health that a person would be expected to live, considering the age-specific mortality and morbidity for a given population in a calendar year (3, 20). We calculated years of life spent with poor health, by age, sex, location, and year, by subtracting healthy life expectancy from life expectancy, and the percentage of years of life spent with poor health as years of life spent with poor health as a percentage of life expectancy (i.e. % of years of life spent with poor health = [life expectancy–healthy life expectancy]/life expectancy × 100). We also analyzed disability-adjusted life years (DALYs), years lived with disabilities (YLDs), and years of life lost (YLLs) due to premature mortality. YLDs represent non-fatal conditions and describe the years lived in less than optimum health, measured as the product of the prevalence estimate and the disability weight for each mutually exclusive condition, corrected for co-morbidities (15, 21). YLLs were calculated by multiplying the number of deaths caused by a disease in each age group by the standard life expectancy at that age, regardless of sex (22). The DALY is a summary measure of total health loss, calculated by adding YLDs and YLLs. Total health loss (measured in DALYs) is also referred to as the disease burden. These measures are reported as age-standardized rates and are calculated by the direct method using the world standard population. We extracted mean estimates and 95% uncertainty interval (UI) for the outcome measures by age, sex, year, and cause for the Region of the Americas, six subregions, and 38 countries and territories (list of countries in the appendix) from the publicly available online GBD results tool (http://ghdx.healthdata.org/gbd-results-tool).

### Leading causes of morbidity and mortality

To determine the underlying disease patterns that affect the burden of disease in older people, we used disease categories at level 3 of the GBD cause list, both fatal and non-fatal conditions, excluding residual categories. We ranked causes of DALYs, YLLs, and YLDs by sex, age group, and location in 2019, and calculated the percentage change from 1990. Because the distribution of causes of DALYs and YLLs are very similar given the large contribution of YLLs to DALYs at older ages, we present the leading causes of premature mortality using YLLs, and disability using YLDs separately.

## RESULTS

### Life expectancy and healthy life expectancy at 65 years

In the Americas, life expectancy at 65 years increased significantly by 2.1 years, from 17.1 years (95% UI 17.0–17.1) in 1990 to 19.2 years (95% UI 18.9–19.4) in 2019. Healthy life expectancy at 65 years increased by 1.4 years, from 12.2 years (95% UI 10.9–12.4) in 1990 to 13.6 years (95% UI 12.2–14.9) in 2019. However, the percentage of years of life spent with poor health remained about the same: 28.8% (95% UI 21.7%–35.8%) in 1990 and 29.0% (95% UI 23.2%–35.7%) in 2019. Indeed, almost one third of life expectancy is spent in poor health, with the gradient increasing with age. Life expectancy and healthy life expectancy increased in all subregions and the percentage of years of life spent with poor health remained constant, except in Central Latin America where it decreased, particularly at 85+ years (Table S1 and Figure S1 in the appendix). Table S1, panel A in the appendix shows a similar analysis for life expectancy and healthy life expectancy at birth.

**FIGURE 1.**
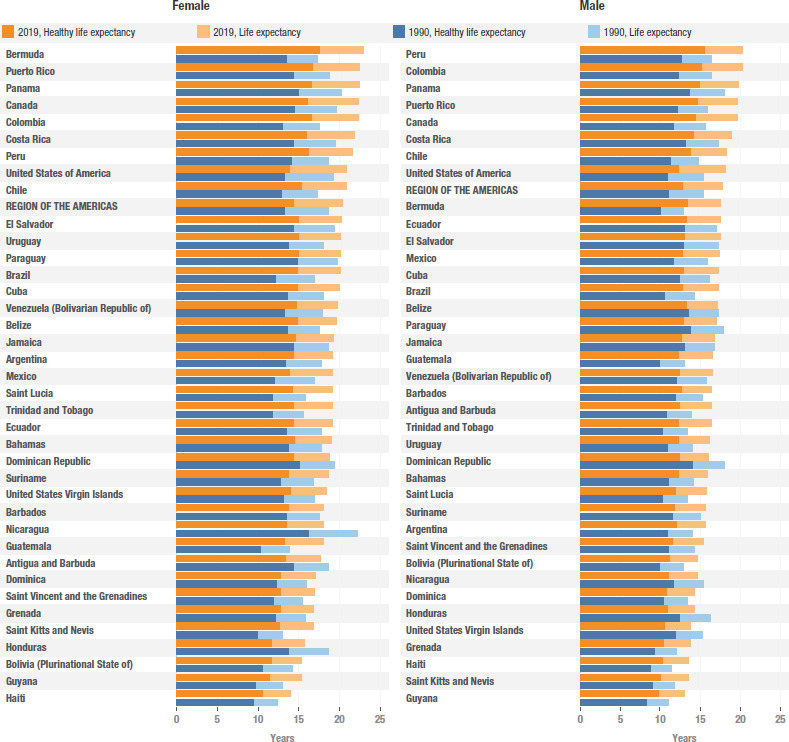
Life expectancy and healthy life expectancy at 65 years of age by country and sex, Region of the Americas, 1990 and 2019

***Source*:** Based on results obtained from the analysis of estimates from the Global Burden of Disease Study 2019. Countries are presented in descending order of rates of disability-adjusted life years (DALY) per 100 000 population in 2019 in each sex separately. The combination of life expectancy measures and years is color-coded as presented in the legend.

Between 1990 and 2019, life expectancy at 65 years substantially decreased in Antigua and Barbuda, the Dominican Republic, Honduras, and Nicaragua for women, and the Dominican Republic, Honduras, Nicaragua, Paraguay, and the US Virgin Islands for men (Figure 1). In 2019, substantial disparities persisted across countries in both life expectancy and healthy life expectancy (Figure 1). Life expectancy and healthy life expectancy at 65 years increased in seven countries (Bolivia, Brazil, Colombia, Costa Rica, Mexico, Paraguay and Venezuela) for women and in four countries (Brazil, Colombia, Mexico and Venezuela) for men. However, in most countries, life expectancy increased but healthy life expectancy decreased. Life expectancy declined and healthy life expectancy increased in only two countries (Nicaragua and Honduras) for women. Both life expectancy and healthy life expectancy declined in two countries for women (Dominican Republic and Antigua and Barbuda) and in six countries for men (Belize, Dominican Republic, Honduras, Nicaragua, Paraguay, and United States Virgin Islands) (Figure S2 in the appendix)

### Burden of disease

**FIGURE 2.**
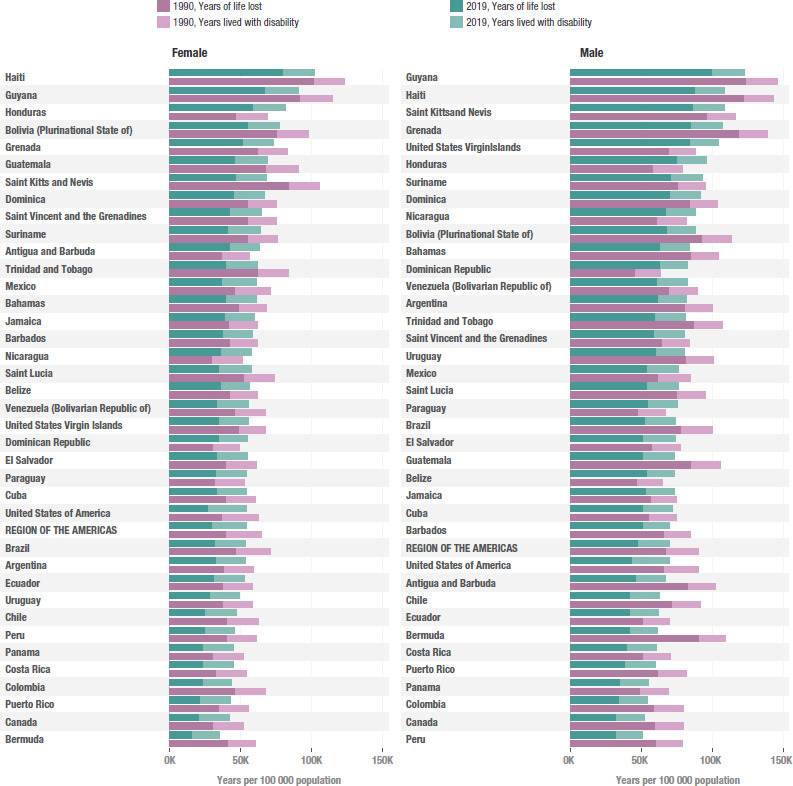
Burden of disease measured by rates of disability-adjusted life years (DALY) per 100 000 population in people aged 65–69 years, broken down as years of life lost and years lived with disability by country and sex, Region of the Americas, 1990 and 2019

***Source:*** Based on results obtained from the analysis of estimates from the Global Burden of Disease Study 2019. Countries are presented in descending order of DALY rates per 100 000 population in 2019 in each sex separately.

From 1990 to 2019, all-cause DALY rates at 65–69 years decreased in most countries except in: Dominican Republic, Honduras, Nicaragua, and Paraguay for both men and women; Antigua and Barbuda for women; and Belize and US Virgin Islands for men (Figure 2). Among countries with DALY rates higher than the regional rate, there are countries with large populations such as Mexico and the USA, high-income countries such as Barbados and Trinidad and Tobago, and Caribbean and Central American countries (Figure 2). In this period, the proportion of all-cause DALYs increased from 22% to 32% in people aged 65 years and over (Figure S3 in the appendix) showing a shift towards old ages, while age-standardized DALY rates for all causes decreased in each older persons’ age group (Figure S4 in the appendix). Figure S4 shows the time trends of age-standardized DALY rates for all causes in both sexes combined at regional and national levels, respectively.

### Premature mortality

Premature mortality is consistently higher in men than women with large differences across countries. YLL rates in people aged 65–69 years in both sexes combined vary from 83 503 years (95% UI 66 227–109 021) in Haiti to 26 703 years (95% UI 26 345–27 079) in Canada (Figure 2). Most countries have a decreasing or constant trend over time in age-specific YLL rates from all causes of mortality, in both sexes in older people; however, six countries (Dominican Republic, Honduras, Nicaragua, Paraguay, Saint Lucia, and Saint Vincent and the Grenadines) have an increasing trend. Regionally, the gradient in premature mortality due to all causes in older adults increases with age, however, with a decreasing time trend at each age regardless of sex (Figure S5, S6, and Table S2 in the appendix).

**FIGURE 3.**
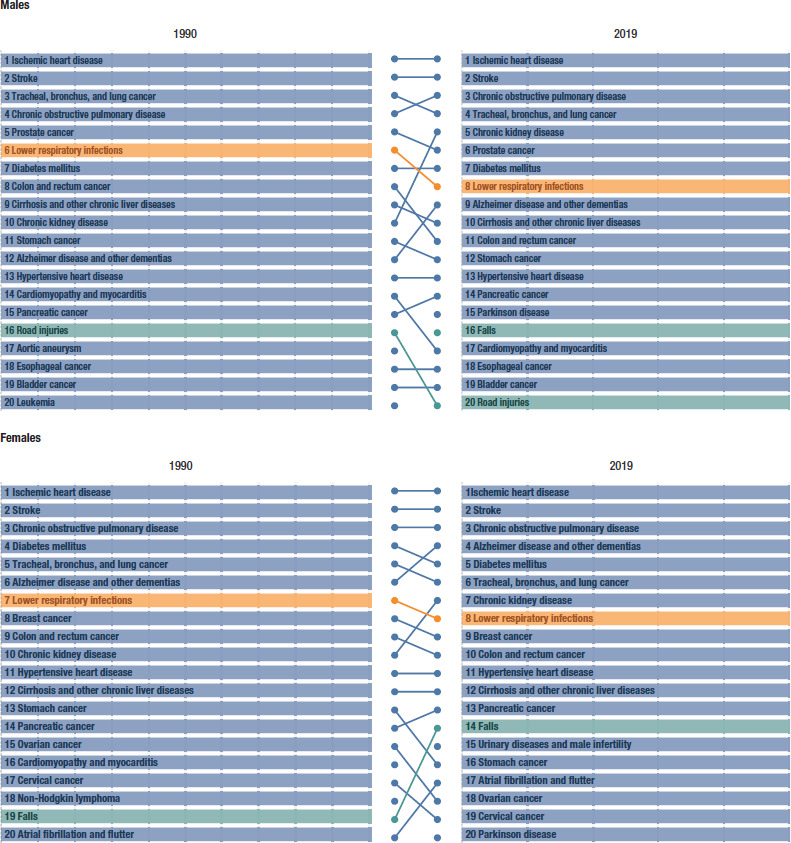
Leading causes of years of life lost due to premature mortality among older people (aged 65 years and older) by sex, Region of the Americas, 1990 and 2019

***Source:*** Based on results obtained from the analysis of estimates from the Global Burden of Disease Study 2019. Causes are color-coded by Level 1 cause category of the Global Burden of Disease Study – orange: maternal, neonatal and communicable diseases; blue: noncommunicable diseases; and green: injury.

In the Americas in 2019, the leading causes of premature mortality among people aged 65+ years for both sexes combined were ischaemic heart disease, stroke, and chronic obstructive pulmonary disease. Lung cancer, chronic kidney disease, prostate cancer, and diabetes mellitus ranked fourth to seventh for men (Figure 3, panel A), and Alzheimer disease and other dementias, and diabetes mellitus ranked fourth and fifth for women, followed by lung cancer, chronic kidney disease, lower respiratory infections, and breast cancer (Figure 3, panel B). Cirrhosis and other chronic liver diseases, and colon and rectum cancer are among the top 20 causes of premature mortality for each sex. From 1990 to 2019, a few changes were observed in the ranking of causes of YLLs due to premature death: chronic kidney disease moved up from tenth to fifth position in men and from tenth to seventh in women, Alzheimer disease and other dementias moved up from twelfth to ninth position in men and from sixth to fourth in women, while falls moved up from 19th position to 14th in women (Figure 3, panels A and B). Parkinson disease, urinary disease, and atrial fibrillation and flutter emerged among the top 20 causes of premature mortality in both sexes combined (Figure S7 in the appendix).

**FIGURE 4.**
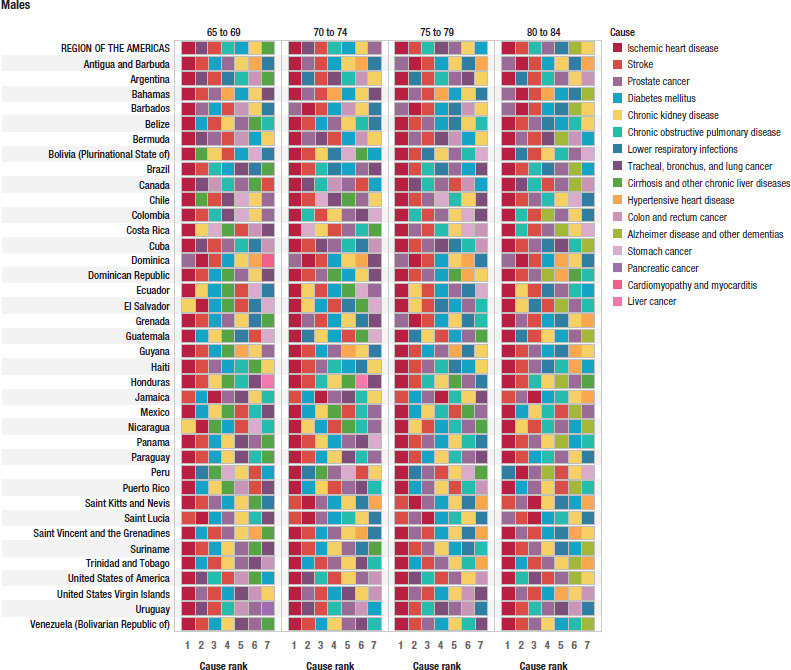

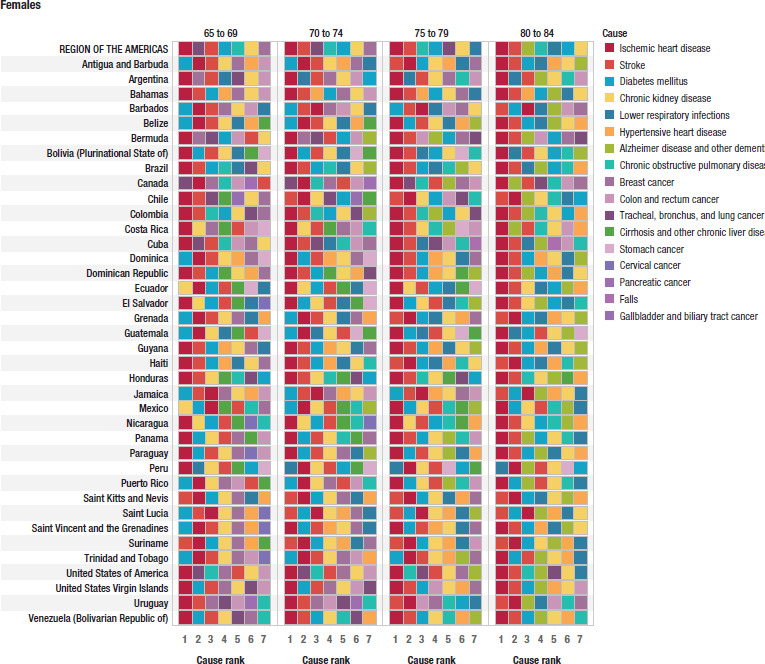
Leading causes of premature mortality by country, age group, and sex, Region of the Americas, 2019

***Source:*** Based on results obtained from the analysis of estimates from the Global Burden of Disease Study 2019.

By country, age, and sex in 2019, ischemic heart disease, stroke, diabetes mellitus, hypertensive heart disease, and chronic kidney disease were the leading causes of YLL. In addition, prostate cancer in men, breast cancer in women, and tracheal, bronchus and lung cancer in both sexes were also leading causes (Figure 4, Figure S8 in the appendix).

### Disability

The burden of disability in older people increased with age and over time; however, YLD per population remained constant or increased slightly in each age group regardless of sex (Figure S9 in the appendix). Between 1990 and 2019, all-cause YLD per 100 000 population for both sexes combined ranged from 23 294 years (95% UI 17 640–29 636) to 23 351 years (95% UI 17 645–29 734) among people aged 65–69 years, and from 36 489 years (95% UI 27 879–45 825) to 37 134 years (95% UI 28 444–46 390) per 100 000 population among people aged 85 years and older. No differences were observed in the level and trends in YLD by sex (Table S2 and Figure S10 in the appendix). In 2019, YLDs in both sexes combined varied across all countries in the Region, from 26 070 years (95% UI 19 823–32 722) in the USA to 18 952 years (95% UI 14 133–24 475) in Bermuda (Figure 2, Table S2 in the appendix).

**FIGURE 5.**
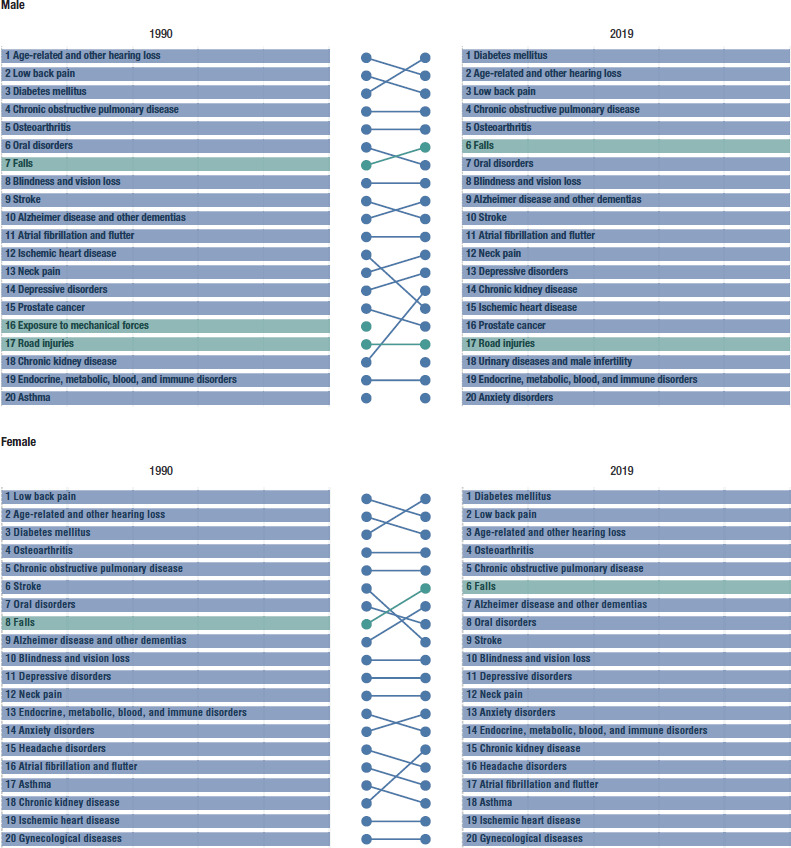
Leading causes of years lived with disability among older people (aged 65 years and older) by sex, Region of the Americas, 1990 and 2019

***Source:*** Based on results obtained from the analysis of estimates from the Global Burden of Disease Study 2019. Causes are color-coded by Level 1 Global Burden of Disease cause category – blue: noncommunicable diseases; green: injury. Any cause from the GBD cause group I Maternal, neonatal and communicable diseases ranked 20th in causes of years lived with disability.

In 2019, most of the leading causes of YLDs among older people were NCDs, but two injuries (falls, and road injuries) were also among the leading causes. The pattern of causes of YLDs in 1990 and 2019 remained constant. In 2019, diabetes mellitus, age-related hearing loss, lower back pain, osteoarthritis, chronic obstructive pulmonary disease, and falls were the top six causes of YLD for men and women (Figure 5). Between 1990 and 2019 in both sexes, diabetes mellitus moved up from third position as a cause of YLD to first. In men, diabetes mellitus, falls, Alzheimer disease and other dementias, neck pain, depressive disorders, and chronic kidney disease all moved up (chronic kidney disease climbed four positions). In women, diabetes mellitus, falls, Alzheimer disease and other dementias, and chronic kidney disease all moved up (Figure 5, Figure S11 in the appendix).

**FIGURE 6.**
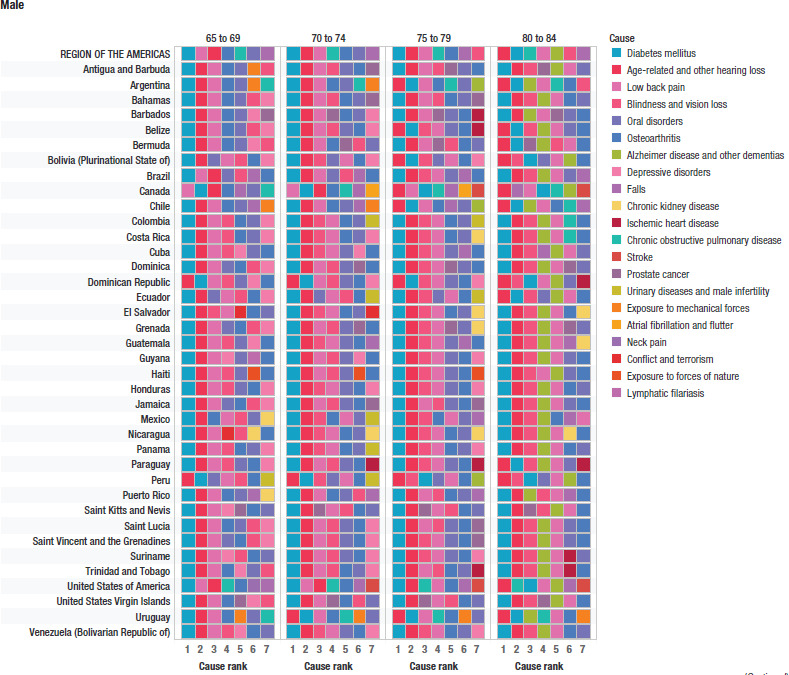

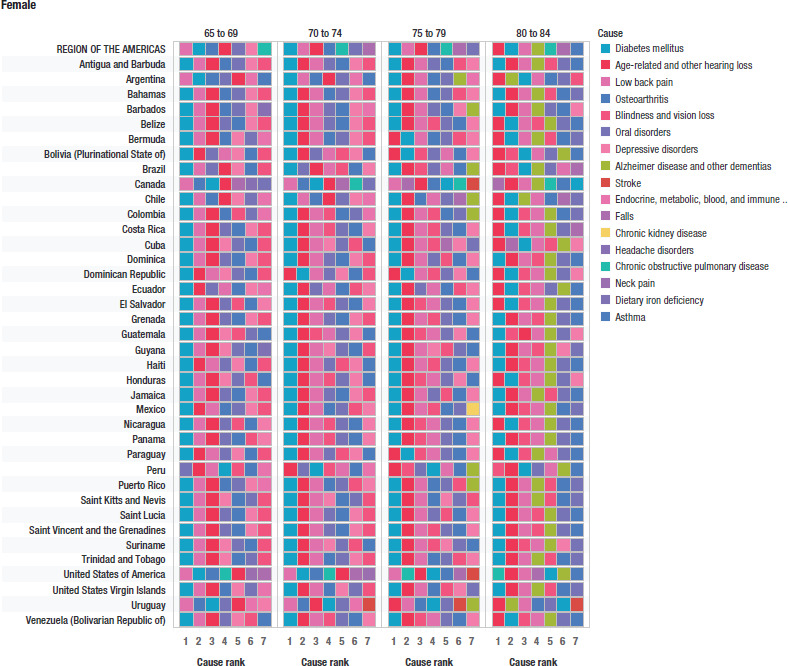
Leading causes of years lived with disability by country, age group, and sex, Region of the Americas, 2019

***Source:*** Based on results obtained from the analysis of estimates from the Global Burden of Disease Study 2019.

In 2019, diabetes mellitus, and age-related and other hearing loss ranked first and second as causes of disability in most countries in both sexes and all age groups 65 years and older. Lower back pain, and blindness and vision loss were also common in most countries and in most age groups (Figure 6, Figure S12 in the appendix).

Life expectancy and YLL in older people had a significant negative association across countries – if YLL increased, then life expectancy decreased – and the values in both measures decreased with age. The percentage of years of life spent with poor health and YLD rates were positively associated at all ages and in both sexes. The values of both measures increased with age and both measures varied substantially across countries (Figure S13 in the appendix).

## DISCUSSION

The Decade of Healthy Ageing is an opportunity to address the health of older adults. Improvement and maintenance of functional ability through strategies to reduce risk factors and provide high-quality health services to prevent and manage NCDs is essential (23). Health at older ages does not mean being disease-free. Instead, healthy aging in the presence of diseases reflects a focus on living well and optimizing functional ability, with the understanding that NCDs can substantially affect an individual’s mental and physical capacities (23).

In the Region of the Americas, age-standardized DALY rates from all causes in the total population decreased over time; however, the distribution of disease burden is shifting to older ages. The consistent decreasing time trends in DALY rates among older people at each age are the result of a substantial reduction in premature mortality, mainly from cardiovascular diseases, the leading cause of death in most countries of the Americas (24). However, a recent study suggests that the decline in premature mortality from cardiovascular diseases has plateaued and even started to increase in the most populated countries of the Region, including the USA and Canada which had previously achieved the most significant reductions (25). This observation is of concern because of the potential effect of the cardiovascular disease burden on life expectancy and healthy life expectancy, especially in people aged 65+ years in the years to come.

In terms of causes of premature mortality, chronic kidney disease, which has shown the highest increase since 1990, deserves special attention. People with chronic kidney disease require specialized and costly health care services. Furthermore, chronic kidney disease is largely preventable, so more efforts are needed to reduce its risk factors and improve care (26, 27). Regionally, the disparity in premature mortality between countries, e.g. mortality from cardiovascular disease (25), has also been substantially reduced. This reduction seems to be associated with a general improvement in the level of economic development and access to and quality of health services. However, it is alarming that some countries with relatively good economic performance in the past decade, such as the Dominican Republic, have shown a decline in life expectancy and an increase in the DALY rate among older people. This decline should be further investigated to understand the potential causes.

The lower rate of increase of healthy life expectancy than life expectancy at 65 years of age in the past three decades means that people are living longer but are spending more of their life time with disabilities and illness. The disability burden revealed underlying conditions that accounted for years of life spent with illness. These conditions ultimately affect quality of life, increase dependence on care, limit the social contribution and opportunities of people of old age (28), and increase the burden on the family and the health system. This finding highlights the importance of providing timely strategies to prevent disability associated with chronic diseases as populations are living longer. In addition, it indicates the urgent need to provide age-disaggregated data in the older adult population and address the oldest age group as a specific group for interventions and research (23).

Diabetes mellitus was the leading cause of YLDs for older people in each age group across countries of the Region in 2019. However, diabetes mellitus is a preventable condition, which is strongly associated with obesity and the obesogenic environment before 65 years. It is also a very manageable clinical condition with good access to and quality of care at all ages, including older ages (29). Indeed, hypertension control combined with cardiovascular disease secondary prevention, which are comprehensive and cost-effective clinical interventions, are still poorly implemented across the world but have enormous potential to reduce both the cardiovascular disease and diabetes mellitus burdens (30, 31).

Apart from the effects of healthy behaviors and personal lifestyle in preventing and managing the main NCDs, physical activity can provide a wide range of improvements in the health of older adults and prevent losses in functional ability (32). For example, falls, a leading cause of disability among older adults in our study, can be reduced by promoting physical activity in a safe environment, especially activities that target different areas (strength, balance and functional exercises) (33).

Age-related and other hearing loss was the second leading cause of disability in our study, which is consistent with other studies (34). Older people with hearing loss are likely to benefit from timely clinical attention, rehabilitation services, and interventions such as hearing aids (35). Sensory health, including hearing and vision capacities, is one of the main domains of intrinsic capacity (physical and mental capacities that an individual can draw on at any point in their life) (36), which is a fundamental aspect of healthy aging.

The burden of disability in older adults, characterized by the presence of more than one chronic condition (multimorbidity) and polypharmacy, together with accelerated aging and the epidemiological transition towards NCDs, pose new health challenges. These factors lead to complex clinical situations that require: innovative health systems; health care capacities to tackle multimorbidity, rather than the single health condition approach; primary health services that are responsive to older people and inter-related with new community roles; and improved access to long-term care for all people who need it (36). The burden of multimorbidity in older people in the Americas should be explored in further studies to guide policy development.

A life-course approach suggests that healthy behavior and lifestyles, access to affordable and good-quality health care, and life opportunities during adulthood can help maintain intrinsic capacities and functional ability after peak capacity has been reached (37). Efforts to improve healthy life expectancy should focus on: building and maintaining intrinsic capacity earlier in life, as its decline is associated with underlying conditions and morbidities, including frailty; reducing time spent in ill-health; and improving older adults’ intrinsic capacity and functional ability (23). A better understanding of how to implement preventive interventions, including self-management, and design sustainable and essential health services for different periods of the life course is needed.

Limitations related to the GBD methodology and data sources for disease burden estimation are described elsewhere (3, 15). A limitation of our study is the under-registration and incomplete medical certification of cause of death in the vital statistics systems of some countries. Moreover, population-based data for morbidity by condition and sequelae are generally insufficient or unavailable for many countries. Estimates for countries with missing or limited data are modeled in the GBD but are of lower reliability than data collected through robust surveillance and vital registration systems, which may explain the large uncertainty for YLD estimates. For morbidity, the GBD study systematically collects all available sources of data, applies standard methods to improve data utility and provide sound estimates. For mortality data, the GBD study addresses these limitations by evaluating data quality. In particular, the GBD study estimates the completeness of death registration, quantifies the proportion of cause-of-death coded as “garbage codes”, applies standard data corrections and adjustments to overcome data incompleteness, and uses death distribution methods to improve the value of underlying cause of death for public health.

In conclusion, the increase in life expectancy and healthy life expectancy at 65 years and decrease of DALY rates in old people over the past three decades in the Americas are probably the result of economic and social progress, the success of public health policies, and improvements in disease prevention and access to and quality of care. However, the small increase in healthy life expectancy relative to the increase in life expectancy shows that, despite living longer, people spend a substantial amount of their old age with disability and illness, which negatively affect their quality of life, increase the burden of care dependency and limit their social contribution.

Despite the decline in disease burden rates among old people in all age groups 65 years and older, its distribution is shifting toward the more older people. Aging and greater longevity are leading to a situation of increased morbidity and disability at old ages.

Preventable or potentially controllable diseases throughout the lifespan are responsible for most of the burden of disease among older people in the Americas. Society-wide and life-course approaches, health investment, and adequate health services are needed to respond to the new health needs of older people, which is critical to achieving the 2030 Agenda for Sustainable Development and for the Decade of Healthy Aging.

## Availability of data and materials.

Data and supplementary materials are available from the corresponding author upon request.

## Disclaimer.

The authors are solely responsible for the views expressed in the manuscript, which may not necessarily reflect the opinion or policy of the Revista Panamericana de Salud Pública/Pan American Journal of Public Health and/or those of the Pan American Health Organization.
